# Interim Estimates of 2021–22 Seasonal Influenza Vaccine Effectiveness — United States, February 2022

**DOI:** 10.15585/mmwr.mm7110a1

**Published:** 2022-03-11

**Authors:** Jessie R. Chung, Sara S. Kim, Rebecca J. Kondor, Catherine Smith, Alicia P. Budd, Sara Y. Tartof, Ana Florea, H. Keipp Talbot, Carlos G. Grijalva, Karen J. Wernli, C. Hallie Phillips, Arnold S. Monto, Emily T. Martin, Edward A. Belongia, Huong Q. McLean, Manjusha Gaglani, Michael Reis, Krissy Moehling Geffel, Mary Patricia Nowalk, Juliana DaSilva, Lisa M. Keong, Thomas J. Stark, John R. Barnes, David E. Wentworth, Lynnette Brammer, Erin Burns, Alicia M. Fry, Manish M. Patel, Brendan Flannery

**Affiliations:** ^1^Influenza Division, National Center for Immunization and Respiratory Diseases, CDC; ^2^Department of Research and Evaluation, Kaiser Permanente Southern California, Pasadena, California; ^3^Vanderbilt University Medical Center, Nashville, Tennessee; ^4^Kaiser Permanente Washington Health Research Institute, Seattle, Washington; ^5^University of Michigan School of Public Health, Ann Arbor, Michigan; ^6^Marshfield Clinic Research Institute, Marshfield, Wisconsin; ^7^Baylor Scott & White Health, Temple, Texas; ^8^Texas A&M University College of Medicine, Temple, Texas; ^9^University of Pittsburgh Schools of the Health Sciences and University of Pittsburgh Medical Center, Pittsburgh, Pennsylvania; ^10^General Dynamics Information Technology, Falls Church, Virginia.

In the United States, annual vaccination against seasonal influenza is recommended for all persons aged ≥6 months except when contraindicated (*1*). Currently available influenza vaccines are designed to protect against four influenza viruses: A(H1N1)pdm09 (the 2009 pandemic virus), A(H3N2), B/Victoria lineage, and B/Yamagata lineage. Most influenza viruses detected this season have been A(H3N2) (*2*). With the exception of the 2020–21 season, when data were insufficient to generate an estimate, CDC has estimated the effectiveness of seasonal influenza vaccine at preventing laboratory-confirmed, mild/moderate (outpatient) medically attended acute respiratory infection (ARI) each season since 2004–05. This interim report uses data from 3,636 children and adults with ARI enrolled in the U.S. Influenza Vaccine Effectiveness Network during October 4, 2021–February 12, 2022. Overall, vaccine effectiveness (VE) against medically attended outpatient ARI associated with influenza A(H3N2) virus was 16% (95% CI = −16% to 39%), which is considered not statistically significant. This analysis indicates that influenza vaccination did not reduce the risk for outpatient medically attended illness with influenza A(H3N2) viruses that predominated so far this season. Enrollment was insufficient to generate reliable VE estimates by age group or by type of influenza vaccine product (*1*). CDC recommends influenza antiviral medications as an adjunct to vaccination; the potential public health benefit of antiviral medications is magnified in the context of reduced influenza VE. CDC routinely recommends that health care providers continue to administer influenza vaccine to persons aged ≥6 months as long as influenza viruses are circulating, even when VE against one virus is reduced, because vaccine can prevent serious outcomes (e.g., hospitalization, intensive care unit (ICU) admission, or death) that are associated with influenza A(H3N2) virus infection and might protect against other influenza viruses that could circulate later in the season.

To derive these interim 2021–22 VE estimates, seven study sites of the U.S. Influenza Vaccine Effectiveness Network (California, Michigan, Pennsylvania, Tennessee, Texas, Washington, and Wisconsin) prospectively enrolled patients aged ≥6 months who had ARI with cough, fever or feverishness, or loss of taste or smell seeking outpatient medical care (i.e., telehealth, primary care, urgent care, or emergency department) or clinical testing for SARS-CoV-2 ≤10 days after illness onset. Inclusion criteria included age ≥6 months on September 1, 2021, enrollment after local influenza circulation was identified,* and no treatment with an influenza antiviral medication (e.g., oseltamivir or baloxavir) during this illness. After informed consent, participants or their guardians were interviewed to collect demographic data, information on general and current health status and symptoms, and 2021–22 influenza vaccination status. A clinical or research upper respiratory specimen for influenza and SARS-CoV-2 molecular testing was collected from eligible patients. Participants who require 2 vaccine doses during their first vaccination season (including children aged <9 years) were considered vaccinated if they received ≥1 dose of any seasonal influenza vaccine ≥14 days before illness onset, according to medical records and registries (Wisconsin site); medical records and self-report (California, Pennsylvania, Tennessee, Texas, and Washington sites); or self-report only (Michigan site). VE against all influenza A viruses and against influenza A(H3N2) viruses was estimated using the test-negative design as 100% x (1 – adjusted odds ratio [OR]).^†^ Using logistic regression, estimates were adjusted for study site, age group, days from illness onset to enrollment, and month of illness onset. This study was reviewed and approved by CDC and U.S. Influenza Vaccine Effectiveness Institutional Review Boards.^§^

Among the 3,636 children and adults with ARI enrolled at the seven study sites during October 4, 2021–February 12, 2022, a total of 194 (5%) received a positive test result for influenza A virus infection by real-time reverse–transcription polymerase chain reaction; none received a positive test result for influenza B virus infection. Among 178 influenza A viruses subtyped, one was A(H1N1)pdm09 and 177 were A(H3N2) viruses (Table 1); 11 patients received positive test results for both influenza A and SARS-CoV-2 viruses. The proportion of patients with influenza differed by study site, age group, and days from illness onset to enrollment. The percentage of ARI patients with reported or documented receipt of 2021–22 influenza vaccine ranged from 31% to 64% among study sites and differed by age group.

**TABLE 1:** Selected characteristics for enrolled patients with medically attended acute respiratory infection, by influenza test result status and seasonal influenza vaccination status* — U.S. Influenza Vaccine Effectiveness Network, United States, October 4, 2021–February 12, 2022

Characteristic	Test result status			Vaccination status*		
	Influenza-positive no. (%)	Influenza-negative no. (%)	P-value^†^	Total no. of patients	Vaccinated no. (%)	P-value^†^
**Overall**	**194 (5)**	**3,442 (95)**	**NA**	**3,636**	**1,817 (50)**	**NA**
**Study site**						
California	3 (1)	438 (99)	<0.001	441	263 (60)	<0.001
Michigan	11 (4)	268 (96)		279	178 (64)	
Pennsylvania	16 (5)	325 (95)		341	147 (43)	
Tennessee	46 (9)	441 (91)		487	251 (52)	
Texas	14 (3)	476 (97)		490	151 (31)	
Washington	4 (1)	405 (99)		409	235 (57)	
Wisconsin	100 (8)	1,089 (92)		1,189	592 (50)	
**Age group**						
6 mos–8 yrs	30 (8)	356 (92)	<0.001	386	214 (55)	<0.001
9–17 yrs	51 (11)	403 (89)		454	163 (36)	
18–49 yrs	87 (5)	1,699 (95)		1,786	793 (44)	
50–64 yrs	19 (3)	653 (97)		672	393 (58)	
≥65 yrs	7 (2)	331 (98)		338	254 (75)	
**Illness onset to enrollment, days**						
<3	112 (6)	1,614 (94)	0.01	1,726	888 (51)	0.28
3–4	55 (5)	1,129 (95)		1,184	578 (49)	
5–7	27 (4)	699 (96)		726	351 (48)	
**Influenza test result**						
Negative	NA	3,442	NA	3,442	1,738 (50)	NA
Influenza A positive	194 (100)	NA		194	79 (41)	
A (H1N1)pdm09	1 (0.5)	NA		1	0 (—)	
A (H3N2)	177 (91)	NA		177	69 (39)	
A subtype pending	16 (8)	NA		16	10 (63)	
Influenza B positive	0 (—)	NA	NA	0	0 (—)	NA

**Abbreviation:** NA = not applicable.

* Defined as having received ≥1 doses of influenza vaccine ≥14 days before illness onset. A total of 101 participants who received the vaccine ≤13 days before illness onset were excluded from the study.

^†^ Pearson’s chi-square test was used to assess differences between the numbers of persons with influenza-negative and influenza-positive test results in the distribution of enrolled patient and illness characteristics and in differences between groups in the percentage vaccinated.

Among participants with a positive influenza test result, 41% had received the 2021–22 seasonal influenza vaccine, compared with 50% of influenza test result–negative participants (Table 2). VE against outpatient medically attended ARI associated with influenza A virus types was 14% (95% CI = −17% to 37%). VE for all ages combined was 16% (95% CI = −16% to 39%) against outpatient medically attended ARI associated with influenza A(H3N2) virus infection.

**TABLE 2:** Number and percentage of persons receiving 2021–22 seasonal influenza vaccine among 3,636 outpatients with acute respiratory infection, by influenza test result status and vaccine effectiveness* against all influenza A and against virus type A(H3N2) — U.S. Influenza Vaccine Effectiveness Network, United States, October 4, 2021–February 12, 2022

Influenza type, all ages	Influenza-positive		Influenza-negative		VE*	
	Total	Vaccinated no. (%)	Total	Vaccinated no. (%)	Unadjusted % (95% CI)	Adjusted % (95% CI)^†^
**Influenza A**	**194**	79 (41)	**3,442**	1,738 (50)	32 (10 to 50)	14 (−17 to 37)
**Influenza A/H3N2**	**177**	69 (39)	**3,174**	1,564 (49)	34 (11 to 52)	16 (−16 to 39)

**Abbreviations:** OR = odds ratio; VE = vaccine effectiveness.

*VE was estimated using the test-negative design as 100% x (1 − OR [ratio of odds of being vaccinated among outpatients who received influenza-positive test results to odds of being vaccinated among outpatients who received influenza-negative test results]); ORs were estimated using logistic regression. https://www.cdc.gov/flu/vaccines-work/us-flu-ve-network.htm

^†^ Adjusted for study site, age group, number of days from illness onset to enrollment, and month of illness using logistic regression.

As of February 12, 2022, CDC had genetically characterized 65 influenza A(H3N2) viruses from U.S. Influenza Vaccine Effectiveness Network participants; all viruses belonged to genetic clade 3C.2a1b subclade 2a.2. This viral subclade has been identified in >99% of genetically characterized A(H3N2) viruses submitted to CDC from U.S. public health laboratories nationwide to date during the 2021–22 influenza season. Post-infection ferret antisera raised against the cell-propagated 2021–22 vaccine reference virus A/Cambodia/e0826360/2020 poorly neutralized the majority of circulating A(H3N2) viruses from subclade 2a.2 (*3*).

## Discussion

This interim estimate of 2021–22 influenza VE suggests that influenza vaccination did not significantly reduce the risk of outpatient medically attended illness with influenza A(H3N2) viruses that have predominated so far this season. These findings are consistent with previous evidence of low to no protection against outpatient infection with A(H3N2) subclade 2a.2 viruses from an investigation of an influenza outbreak on a university campus during October–November 2021 (*4*). These VE estimates underscore the need for ongoing diagnostic testing for influenza, influenza antiviral treatment and prophylaxis when indicated, and everyday preventive measures (*4*,*5*). CDC continues to recommend influenza vaccination when VE against outpatient illness is reduced because a growing body of evidence suggests that influenza vaccination can avert serious outcomes, including hospitalization, ICU admission, and death, among persons who are vaccinated but still become infected (*6*). In addition, vaccination is likely to prevent illness or serious complications of infection with other influenza viruses that might circulate later in the season, including influenza A(H1N1)pdm09 and B viruses (*6*).

Compared with influenza vaccination during 2020–21, influenza vaccination coverage is lower so far this season in certain groups, including some groups who are at high risk for severe influenza or complications from influenza, such as persons who are pregnant, infants, and preschool-aged children, as well as persons from racial and ethnic minority groups (*7*). Persons aged ≥6 months who have not yet been vaccinated this season should be vaccinated.

This influenza VE estimate is the first since the 2019–20 season; effectiveness of 2020–21 influenza vaccines could not be assessed because influenza virus circulation was historically low. Cumulative rates of laboratory-confirmed influenza hospitalizations so far this season have also been substantially lower than in recent A(H3N2)-predominant seasons (*7*). During the 2021–22 influenza season, clinical laboratory data reported to CDC showed increased influenza virus circulation beginning in November 2021 and continuing through mid-December 2021. From late December 2021 through late January 2022, during the rapid rise in SARS-CoV-2 B.1.1.529 (Omicron) variant positivity, influenza activity declined; however, during the first 2 weeks of February 2022, a slight increase in the percentage of specimens testing positive for influenza at clinical laboratories was reported. Influenza activity is difficult to predict and may continue for multiple weeks.

On February 25, 2022, the World Health Organization issued recommendations that the 2022–23 influenza vaccines for the northern hemisphere include updates to A(H3N2) reference viruses representing the 2a.2 subclade of A(H3N2) clade 3C.2a1b, as well as updates to the B/Victoria lineage vaccine component (*3*). Predicting circulation of virus subtypes and predominant clades within subtypes remains challenging. Evolution of circulating viruses has required frequent updates to the composition of influenza vaccines. Efforts to develop influenza vaccines that provide broader coverage of the diversity among circulating viruses are ongoing.

The findings in this report are subject to at least four limitations. First, because of low influenza test positivity, VE estimates were limited to all ages combined against influenza A overall and against A(H3N2); VE can vary by virus type or subtype (*8*), vaccine formulation, and antigenic match between circulating viruses and vaccine components (*9*,*10*).^¶^ End-of-season VE estimates could change as enrollment continues or if other influenza viruses predominate later in the season. Second, vaccination status at six of seven sites included self-report, which might result in misclassification of influenza vaccination status for some patients. Third, health care seeking behavior has changed during the COVID-19 pandemic and enrollment of patients with outpatient illness from COVID-19 testing sites might have affected results. The test-negative design for estimating influenza VE requires validation when influenza test-negative controls include patients with COVID-19 and receipt of influenza and COVID-19 vaccines are correlated. Finally, VE estimates in this report are specific to the prevention of outpatient illness rather than to more severe illness outcomes (e.g., hospitalization or death); data from studies measuring VE against more severe outcomes this season will be available at a later date.

Although influenza virus circulation and laboratory-confirmed influenza associated hospitalizations declined from late December 2021 through January 2022, some regions of the United States have seen increases in influenza activity since that time.** Influenza activity is difficult to predict, and strategies to prevent influenza illness remain important to reduce strain on health care services. Vaccination against seasonal influenza might protect against other influenza viruses that could circulate later in the season and their potentially serious complications. Clinicians should consider diagnostic testing for patients with ARI, especially among hospitalized patients and those at increased risk for complications. All hospitalized patients and all outpatients at higher risk for serious complications from influenza should be treated as soon as possible with a neuraminidase inhibitor medication if influenza is suspected (*5*). Physicians should not wait for confirmatory influenza laboratory testing, and the decision to use antiviral medication should not be influenced by patient influenza vaccination status. Clinicians should be aware that influenza activity might continue or increase, and influenza should be considered as a possible diagnosis in all patients with ARI.

**Summary**

**What is already known about this topic?**

Annual vaccination against seasonal influenza is recommended for all persons in the United States aged ≥6 months. Effectiveness of seasonal influenza vaccine varies by influenza season.

**What is added by this report?**

Based on data from 3,636 children, adolescents, and adults with acute respiratory infection during October 4, 2021–February 12, 2022, seasonal influenza vaccination did not reduce the risk for outpatient respiratory illness caused by influenza A(H3N2) viruses that have predominated so far this season.

**What are the implications for public health practice?**

CDC recommends influenza vaccination for as long as influenza viruses are circulating. Vaccination can prevent serious influenza-related complications caused by viruses that might circulate later in the season, including 2009 pandemic A(H1N1) and influenza B viruses.

## Footnotes

* U.S. Influenza Vaccine Effectiveness Network sites and the dates of the first influenza-positive case by site are the University of Michigan School of Public Health (in partnership with University of Michigan Health System [Ann Arbor, Michigan] and the Henry Ford Health System [Detroit, Michigan]) (October 4, 2021); Vanderbilt University Medical Center, (Nashville, Tennessee) (November 2, 2021); Kaiser Permanente Washington (Seattle, Washington) and Kaiser Permanente Southern California (Los Angeles, California) (November 9, 2021); Baylor Scott & White Health (Temple, Texas) (November 21, 2021); Marshfield Clinic Research Institute (Marshfield, Wisconsin) (November 24, 2021); and University of Pittsburgh Schools of the Health Sciences (in partnership with the University of Pittsburgh Medical Center, Pittsburgh, Pennsylvania) (November 29, 2021).

† 100% x (1 − OR [ratio of odds of being vaccinated among outpatients who received positive test results to CDC’s real-time reverse–transcription polymerase chain reaction influenza test to the odds of being vaccinated among outpatients who received influenza-negative test results]).

§ 45 C.F.R. part 46; 21 C.F.R. part 56.

¶ Sample sizes to achieve an adequate number of influenza cases to estimate a significant VE with 95% CIs that do not include 0 were estimated for the following age groups: 6 months–17 years, 18–49 years, and ≥50 years. Sample size calculations were based on a type I error probability of 5% and a type II error probability of 20% (power 80%) to detect 40% VE against any influenza and 30% VE against influenza A(H3N2). Assumptions about vaccination coverage varied by age group: 50% for 6 months–17 years, 30% for 18–49 years, and 50% for ≥50 years.

**
https://www.cdc.gov/flu/weekly/index.htm
